# Electrochemical noise and impedance of Au electrode/electrolyte interfaces enabling extracellular detection of glioma cell populations

**DOI:** 10.1038/srep34843

**Published:** 2016-10-06

**Authors:** Paulo R. F. Rocha, Paul Schlett, Ulrike Kintzel, Volker Mailänder, Lode K. J. Vandamme, Gunther Zeck, Henrique L. Gomes, Fabio Biscarini, Dago M. de Leeuw

**Affiliations:** 1 Max Planck Institute for Polymer Research, Ackermannweg 10, D-55128 Mainz, Germany; 2 Dermatology Clinic, University Medicine of the Johannes-Gutenberg Universität Mainz, Langenbeckstrasse 1, 55131 Mainz, Germany; 3 Electrical Engineering, Eindhoven University of Technology, P.O. Box 513, 5600MB Eindhoven, Netherlands; 4 Natural and Medical Sciences Institute, Markwiesenstrasse 55, D-72770 Reutlingen, Germany; 5 IT-Instituto de Telecomunicações, Av. Rovisco, Pais, 1, 1049 - 001 Lisboa, Portugal; 6 Universidade do Algarve, FCT, 8005-139 Faro, Portugal; 7 Life Science Department, Università di Modena e Reggio Emilia, Via Campi 103, I-41125 Modena, Italy

## Abstract

Microelectrode arrays (MEA) record extracellular local field potentials of cells adhered to the electrodes. A disadvantage is the limited signal-to-noise ratio. The state-of-the-art background noise level is about 10 μVpp. Furthermore, in MEAs low frequency events are filtered out. Here, we quantitatively analyze Au electrode/electrolyte interfaces with impedance spectroscopy and noise measurements. The equivalent circuit is the charge transfer resistance in parallel with a constant phase element that describes the double layer capacitance, in series with a spreading resistance. This equivalent circuit leads to a Maxwell-Wagner relaxation frequency, the value of which is determined as a function of electrode area and molarity of an aqueous KCl electrolyte solution. The electrochemical voltage and current noise is measured as a function of electrode area and frequency and follow unambiguously from the measured impedance. By using large area electrodes the noise floor can be as low as 0.3 μVpp. The resulting high sensitivity is demonstrated by the extracellular detection of C6 glioma cell populations. Their minute electrical activity can be clearly detected at a frequency below about 10 Hz, which shows that the methodology can be used to monitor slow cooperative biological signals in cell populations.

Extracellular activity of electrogenic cells is commonly recorded using microelectrode arrays (MEAs) which consists of planar electrodes on a substrate in close contact with cells in culture medium. MEAs detect the extracellular field potential, which is a superposition of *e.g.* fast action potentials of individual neurons, through synaptic potentials, to glial potentials slowly varying in time and space. The first MEA was demonstrated in the early 70’s^1^. Since then, research has been focused to improve spatial resolution and electrical coupling between the cell and the sensing electrodes^2^,^3^,^4^,^5^,^6^,^7^. The spatial resolution has been improved by increasing the density and the number of the electrodes. Arrays with 10^4^ electrodes with area of 30 μm^2^, each only tens of microns apart have been reported^8^,^9^.

Extracellular voltages are strongly attenuated with respect to the intercellular voltages. The attenuation factor is in between 0.01 and 0.001. The extracellular voltages are typically between 10 μV and 1 mV. To detect these small voltages the background noise should be as low as possible. The background noise is due to the instrumentation noise and the electrochemical noise of the electrode/electrolyte interface. For metal-based electrodes the latter is directly proportional to the real part of the impedance. Hence, impedance is being minimized by judicious choice of electrode materials, such as modification with porous conductive materials such as Pt-black, carbon nanotubes and conducting polymers^10^. State-of-the-art MEA systems exhibit a background noise of about 10 μVpp^10^.

The main application of MEAs is detection of fast events such as action potentials. Traditionally, these signals are measured using voltage amplifiers with a bandwidth of at least 1 kHz. Low frequency neuronal oscillations are filtered out; their detection is impaired or even inhibited. However, detection of low frequency activity is crucial to understand brain physiology as changes in low frequency neuronal oscillations have been associated with brain disorders such as schizophrenia and epilepsy.

To optimize the signal to noise ratio (SNR) the electrode impedance should be as small as possible as compared to the amplifier input impedance. It is a challenge to achieve a low impedance with micrometer size, plain electrodes. Therefore, research is focused on increasing the effective surface area by modifying the electrode with porous conducting materials such as Pt black, Au nanostructures and carbon nanotubes. Here, however, we take a different approach. We decrease the impedance by using extremely large electrode areas of a few mm^2^, orders of magnitude larger than electrode areas used in conventional MEA systems. The electrolyte/electrode interface is characterized by impedance spectroscopy. The impedance as a function of frequency and electrode area is quantitatively analyzed. The power spectral density of the current and voltage noise agrees with the measured electrode impedance. We show that the electrochemical noise floor can be as low as 0.3 μVpp by using electrode areas up to a few mm^2^.

The breakthrough sensitivity is demonstrated by extracellular recordings of glioma cells. Glia cells, as well as their transformed counterpart glioma cells, are non-electrogenic cells that do not exhibit action potentials. However, they exhibit fluctuations in membrane potential. The electrophysiology such as voltage gated ion channels has been investigated by patch clamp measurements. Extracellular MEA recordings are scarce as the attenuated extracellular potentials are too weak. Here, we show that the ultra-low background noise allows the detection at low frequency of the minute electrical activity of glioma cells. As a model system we used rat glioma C6 cells. The activity is recorded in current and voltage. However, due to the large electrode area, single cells adhered to the electrode cannot be addressed. The spatial information is lost. The measured signal is the sum of all individual cell contributions. When the activity of the cells is not coordinated, the overall signal of the whole population appears as uncorrelated noise. We show that the electrical activity is measured when the cells are highly viable and proliferate. The electrical activity has been correlated with the density of glioma cells adhered to the sensing electrode.

## Experimental

The transducer consists of a glass substrate on which circular electrodes of 100 nm Au on top of a 4 nm Cr adhesion layer were evaporated through a shadow mask. The surface roughness as measured with a DEKTAK surface profilometer amounted to less than 1 nm. Hence the electrode area can be taken equal to the geometrical value of the circular electrodes. The areas used amounted to 2 mm^2^, 6 mm^2^ and 10.5 mm^2^. Then a drilled PMMA well is glued on top of the substrate with the prefabricated electrodes. This well serves as a container for the electrolyte solution or for the C6 glioma cell suspension. The circular electrodes are located inside the well and connected with a small strip-line to the contact pad outside the well. Hence the impedance and noise measurements can be performed by connecting the contact pads outside the electrolyte solution. The area of the strip-line can be disregarded with respect to the area of the electrodes.

As electrolyte an aqueous KCl solution of varying molarity between 10^−4^ M and 1 M was used. The small signal impedance was measured between pairs of identical electrodes at zero applied bias with a Solatron SI 1260 impedance analyzer in the frequency range of 1 Hz to 1 MHz. At the start of the measurements small drifts in current and in voltage were observed. However, after about 20 minutes the recording system stabilizes. All data presented here were measured after this incubation time, and the data has not been corrected for any slowly varying background.

Current and voltage noise measurements were performed by a Stanford low-noise current amplifier (SRS 570) and a Brookdeal 5003 low noise voltage amplifier. All electric noise measurements were unbiased. The noise spectra were analyzed with an Agilent 35670A spectrum analyzer in 3 frequency spans, i.e. 0.5–100 Hz, 16 Hz–3.2 kHz and 512 Hz–51.2 kHz, in order to present spectral values from 0.5 Hz up to 51.2 kHz. All frequency spans represent an average of at least 20 continuous recordings.

Here we focus on bio-electronic signals at low frequency, below 10 Hz. The signals are weak. To optimize the signal to noise ratio we use the highest possible amplification with the bandwidth as low as 20 Hz. The time resolution is then sufficient to resolve slow oscillations in the extracellular field potentials. The ultrasensitive detection system was realized using a current amplification of 5 nA/V and a voltage gain of 1000. External interference was minimized through the use of a Faraday cage and low noise cables.

Rat glioma C6 cells (American Type Culture Collection, ATCC) were cultured in 15% F-12K nutrient mixture supplemented with 15% of fetal horse serum, 2.5% of fetal bovine serum and 1% of penicillin and streptomycin. The adherent glioma cells were maintained at 37 °C in an incubator with a humidified atmosphere with 5% of CO_2_. Upon cell detachment using trypsin/EDTA, cell numbers and viability were assessed using a Neubauer chamber-based trypan blue live/dead exclusion assay. The cells were immersed in the PMMA well. An aliquot of 1 ml of C6 glioma cell suspension with a concentration of 1 × 10^6^ cells/ml was transferred in to the well. The cells were allowed to adhere to the electrodes for 2 h before any measurements were performed.

### Impedance spectroscopy of the Au electrode/electrolyte interface

**Figure 1:**
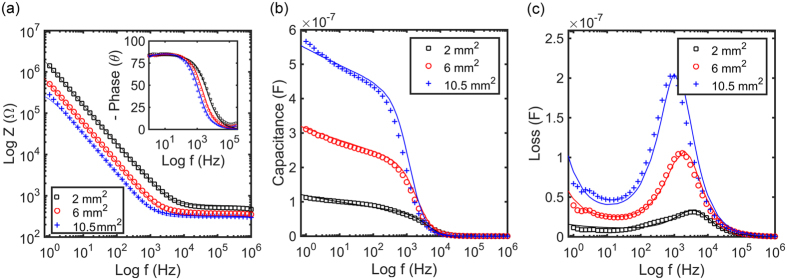
Small-signal impedance of Au electrodes in 100 mM aqueous KCl solution. The electrode area was varied between 2 mm^2^, 6.5 mm^2^ and 10.5 mm^2^. (**a**) Impedance as a function of frequency between 1 Hz and 1 MHz. The inset shows the corresponding phase angle. (**b**) Extracted equivalent parallel capacitance, *C*_*p*_. (**c**) Extracted dielectric loss, *G*_*p*_*/ω*. The peak in the dielectric loss corresponds to the Maxwell-Wagner relaxation frequency. The solid lines are fits derived by fitting the impedance using Eqs [3 and 4]. The equivalent circuit is presented in the inset of Fig. 2a.

Figure 1a shows the small-signal impedance as a function of frequency between 1 Hz and 1 MHz. The inset shows the corresponding phase angle. Measurements were performed in 100 mM aqueous KCl solution for different electrode areas. This molarity was chosen as the conductivity of the electrolyte then mimics the conductivity of a standard cell culture medium. The extracted equivalent parallel capacitance, *C*_*p*_, is presented in Fig. 1b. At low frequency the capacitance is not constant but slightly decreases with increasing frequency. At about 1 kHz the capacitance decreases to a negligible value. The relaxation frequency that characterizes the dispersion in capacitance can be obtained from the maximum in the corresponding dielectric loss spectrum. The loss, defined as the equivalent parallel conductance, *G*_*p*_, over the angular frequency, *ω*, is presented as a function of frequency in Fig. 1c. A maximum is observed that slightly depends on the electrode area.

**Figure 2:**
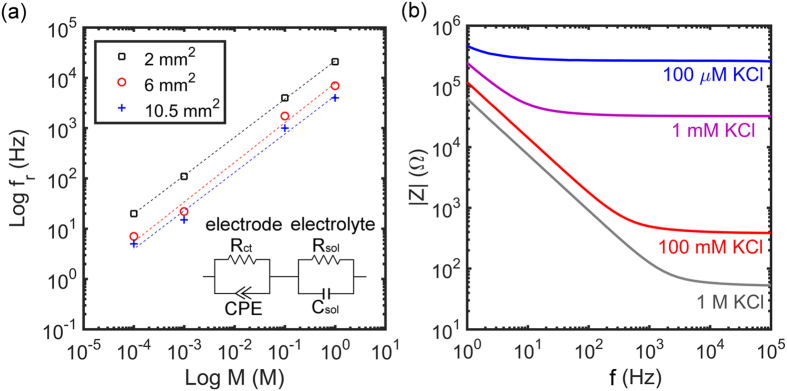
Impedance as a function of molarity. (**a**) Maxwell-Wagner relaxation frequency as a function of molarity of an aqueous KCl solution. The frequency is extracted for the three different electrode areas. The dotted lines are a power law fit to the data with slope 0.9. Inset depicts the Randles equivalent circuit of an electrode-electrolyte interface, consisting of an interface capacitance, described by a constant phase element, CPE, shunted by a frequency independent charge transfer resistance, *R*_*ct*_, in series with the solution resistance, *R*_*sol*_. The negligible solution capacitance, *C*_*sol*_, can be disregarded. (**b**) Impedance as a function of molarity of the aquous KCl electrolyte solution.

The observed dispersion in capacitance is due to the Maxwell-Wagner relaxation effect, which is an interfacial relaxation process that occurs in all systems where electrical current must pass an interface between two dielectrics. This relaxation effect can be described by the Randles equivalent electrical circuit as presented in the inset of Fig. 2a. The equivalent circuit is a series-parallel network of the electrode interface and the electrolyte solution. We note that as both electrodes are equal, only one electrode interface has to be included.

At equilibrium, equal, and opposite reduction and oxidation currents flow across the electrode-electrolyte interface. For an ideally polarizable, or blocking, electrode this exchange current density is zero, and for an ideally unpolarizable electrode this current density tends to infinity, limited by the instrumental series resistance. Here we use an Au electrode which behaves as an almost ideally polarizable electrode. The equilibrium exchange current density depends exponentially on applied bias. However, at low bias the exchange current density follows Ohms law. The Faradaic redox processes can then be characterized by a frequency independent, linear charge transfer resistance, *R*_*ct*_.

At any interface an electrical double layer is formed. This double layer is also present at an electrode electrolyte interface. The corresponding capacitance can be described in the Gouy-Chapman-Stern model as a series capacitance of the Helmholtz double layer capacitance and the Gouy-Chapman diffuse layer capacitance. The value of the capacitance depends on the applied potential and the ionic strength of the solution. Only for ideally polarizable electrodes, such as Hg, the double layer capacitance can be represented by a linear capacitor. In all other cases, the double layer capacitance has to be described by a constant phase element, CPE. Using the IUPAC recommendation, the impedance of the CPE is given as:


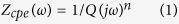


The value of the fractional exponent, *n*, is in between −1 and 1. Q is a frequency independent, phenomenological parameter. When *n* is 1 the impedance simplifies to that of a discrete linear capacitor. The physico-chemical origin of the CPE behaviour at the interface is not yet fully understood. It has been attributed to inhomogeneities of the surface leading to a distribution of time scales^11^,^12^,^13^,^14^,^15^,^16^,^17^,^18^,^19^,^20^ as liquid metal electrodes with a perfect smooth surface show ideal capacive behaviour characterized by *n* equal to 1. Due to the smooth surface, fringe fields can then be disregarded.

The solution resistance is due to the spreading of current from the localized electrode to a distant counter electrode in the solution. For a system with a reference electrode and an infinite counter electrode, the resistance can be calculated. For a circular electrode of radius, *r*, the spreading resistance is given by:


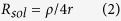


where *ρ* is the specific resistivity of the solution. The approximation is the better the larger the electrode area as the contribution of fringe fields at the rim of the electrode can then be ignored. The solution capacitance, *C*_*sol*_, is negligible small and can be disregarded.

The real and imaginary parts of the impedance, *Z*′ and *Z*″ are then given by:


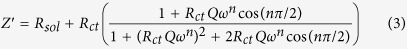



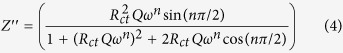


For *n* equal 1, the impedance is equal to that of Randles equivalent circuit consisting of a resistance in series with an RC circuit, *c.f.* inset of Fig. 2. The equivalent parallel capacitance, *C*_*p*_, then is constant. Figure 1b shows that the extracted parallel capacitance increases with decreasing frequency. Hence the impedance has to be described by Eqs [2 and 3] with *n* as a fractional fitting constant. The fully drawn curves in Fig. 1 are fits to the data. A good description is obtained. The fit parameters are given in Table 1.

The origin of the impedance can be understood as follows. At low frequency the impedance is dominated by that of the electrode and at high frequency by that of the series solution. The parallel conductance, *G*_*p*_, approaches at low frequency *1/R*_*ct*_ and at high frequency *1/R*_*sol*_. The charge transfer resistance scales with area. The surface resistance, *R*_*ct*_ *S*, is constant and the harmonic mean of 2.7*10^5^ Ωcm^2^ indicates that the Au electrode used behaves as an almost ideal polarizable electrode.

The spreading resistance scales with area. Table 1 shows that by using Eq. [2], a specific resistivity, *ρ*, for the 100 mM aqueous KCl solution is calculated to be about 200 Ωcm, in fair agreement with the measured Sigma Aldrich reference value.

At low frequency the parallel capacitance is dominated by the double layer capacitance. The frequency dependence follows from the extracted CPE element as *C*_*p*_ is equal to *Qω*^*n–*1^. The value of the exponent *n* of about 0.9 is close to 1 as expected for an almost ideal polarizable electrode. To estimate the double layer capacitance from the CPE values we use the approximation of Hsu^21^:


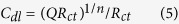


The calculated double layer capacitance scales with area and amounts on average to about 13 μF/cm^2^ (*c.f.* Table 1), in good agreement with literature data^22^. At high frequency the capacitance becomes equal to the capacitance of the solution. The value is too small to reliably extract from the high frequency impedance. The relaxation frequency that characterizes the dispersion in equivalent parallel capacitance can be obtained from the maximum in the corresponding dielectric loss spectrum. The loss, *G*_*p*_*/ω*, is low at both low and high frequency and equal to *1/ωR*_*ct*_ and *1/ωR*_*sol*_ respectively. The maximum loss is obtained at the Maxwell-Wagner frequency, *f*_*r*_, that is given by^23^:


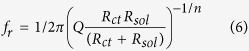


As the extracted charge transfer resistance is orders of magnitude larger than the spreading resistance, the relaxation frequency reduces to:


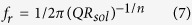


The impedance was measured upon varying the concentration of KCl in the aqueous solution between 100 μM and 1 M and the measured values are presented in Fig. 2b. At low frequency the impedance is dominated by the sensing electrode. At high frequency the charge transfer resistance is shorted by the double layer capacitance, and the impedance is dominated by the electrolyte solution. Hence, when the salt concentration increases, the solution resistance decreases leading to a gradual shift of the Maxwell-Wagner relaxation frequency. The impedance of Fig. 2b has been fitted with Eqs [3 and 4] and a good agreement was obtained.

The extracted relaxation frequency as a function of molarity is presented on a double logarithmic scale in Fig. 2a. For each electrode area a straight line is observed with a slope of 0.9. The capacitance is hardly frequency dependent. At high molarity the capacitance is constant and equal to the Helmholtz double layer capacitance. The contribution of the diffuse capacitance can then be disregarded. The solution resistance depends on the specific conductivity of the aqueous KCl solution, *cf.* Eq. [2], which itself depends linearly on the molarity. Hence the slope of 0.9 of Fig. 2a perfectly agrees with the fitted value of the fractional exponent, *n*, of about 0.9.

### Electrochemical noise of Au electrode/electrolyte system

**Figure 3:**
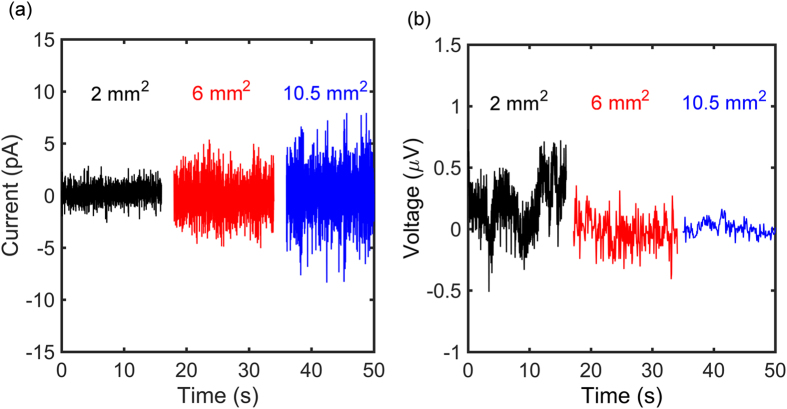
Electrochemical current- and voltage noise as a function of time of unbiased Au electrodes in aqueous 100 mM KCl solution. The electrode areas are 2 mm^2^, 6 mm^2^ and 10.5 mm^2^. The current noise increases with electrode area from 1 pApp to 10 pApp, while the voltage noise decreases with electrode are from 0.7 μVpp through 0.5 μVpp to 0.3 μVpp. The current amplification was 5 nA/V and the voltage gain was 1000.

Electrode-electrolyte systems are used to detect electrical signals of adhered cells. The extracellular bio-electronic signals can be extremely weak. To benchmark the signal to noise ratio that can be obtained, we characterized the electrochemical current and voltage noise of the Au electrode in 100 mM aqueous KCl solution. Electrodes were shorted and the unbiased noise was measured as a function of time. Typical plots of the current and voltage noise with time are presented in Fig. 3. The current noise increases with electrode area from 1 pApp to 10 pApp, while the voltage noise decreases with electrode area from 0.7 μVpp to 0.3 μVpp.

**Figure 4:**
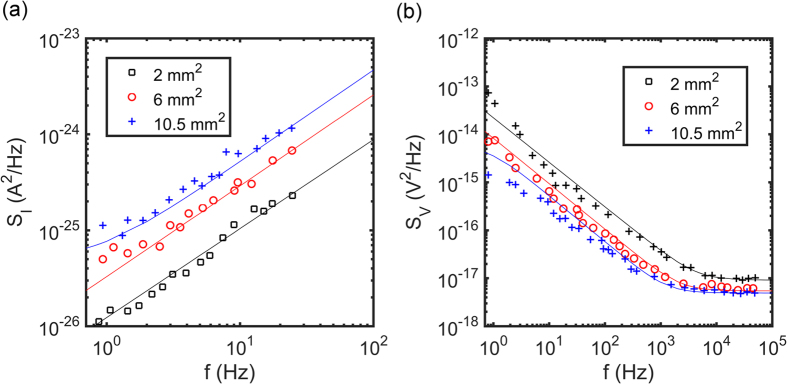
(**a**) The power spectral density of current noise, *S*_*I*_, as a function of frequency. (**b**) The power spectral density of voltage noise, *S*_*V*_, as a function of frequency. The noise was measured unbiased. The noise spectra are averages over 20 consecutive measurements. Different symbols indicate different electrode areas. The measurements were performed in 100 mM aqueous KCl solution. The solid lines are calculated from the measured impedance using Eqs [8 and 9].

To analyze the data we extracted the spectral power density of the noise. The current and voltage fluctuations were recorded in periods of 16 s. For each recording the power spectral density was calculated. The power spectral density of current noise, *S*_*I*_, and voltage noise, *S*_*V*_, are averages over 20 consecutive measurements, and presented as a function of frequency in Fig. 4a,b respectively. The bandwidth of the current amplifier limits the frequency window of the current noise to 20 Hz. To minimize drift, the system was equilibrated for two hours before starting the recordings. We note that various procedures have been reported to correct for slow remaining background oscillations, such as moving average, linear regression and polynomial fitting. These methodologies were not applied as they cancel part of the signal fluctuations and consequently alter the final spectral power density^24^.

In thermodynamic equilibrium, at zero Volt overpotential, the power spectral density of the noise is given by:


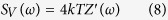



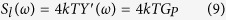


with


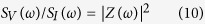


where *k* is the Boltzmann constant, *T* is the absolute temperature, *Z*′ is the real part of the impedance and *Y*′ is the real part of the admittance, equal to the equivalent parallel conductance, *G*_*p*_. The solid lines in Fig. 4 are calculated from the experimentally determined impedances using Eqs [8 and 9]. A good agreement is obtained.

The dispersion in the noise can be explained as follows. We first take a single RC circuit, consisting of a single charge transfer resistance, *R*_*ct*_, in parallel with a double layer capacitance, *C*_*dl*._ The parallel conductance then is constant and equal to one over the charge transfer resistance. The power spectral density of the current noise is then frequency independent and equal to *4kT/R*_*ct*_. The power spectral density of voltage noise is equal to *4kTR*_*ct*_*/*(*1* + (*wR*_*ct*_*C*_*dl*_)^2^). At high frequency, where *ω* *>* *1/R*_*ct*_*C*_*dl*_, the voltage noise reduces to *4kTR*_*ct*_*/*(*wR*_*ct*_*C*_*dl*_)^2^, yielding a *f*-^−2^ frequency dependence. However, here we have a spreading resistance, *R*_*sol*_, in series with the RC circuit. The equivalent parallel conductance changes from *1/R*_*ct*_ at low frequency to *1/R*_*sol*_ at the Maxwell-Wagner relaxation frequency. Consequently, the current noise increases about linear with frequency and the voltage noise decreases about linear with frequency. Similarly, at low frequency both voltage and current noise scales linearly with area as it is dominated by the charge transfer resistance, while at high frequency the noise scales with electrode radius as it is dominated by the spreading resistance. Finally we note that because the noise is calculated using experimentally measured values of the impedance, the fluctuation dissipation theorem holds and Eq. [10] is automatically fulfilled.

### Electrical detection of C6-glioma cells

The use of large electrodes with low impedance electrodes leads to an extremely low background noise level. Here we demonstrate the resulting high sensitivity by the detection of extracellular voltages of glioma cell populations. In order to use glioma cells as a model system we first investigated cell density and viability.

**Figure 5:**
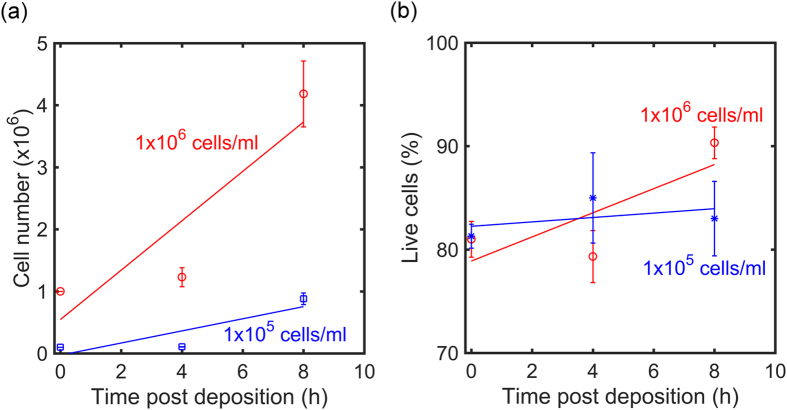
Glioma cell growth and viability. (**a**) Cell number as a function of time after cell deposition for a cell seeding density of 1 × 10^5^ cells/ml in blue and a cell seeding density of 1 × 10^6^ cells/ml in red. (**b**) Corresponding cell vitality as a function of post deposition time. Each data point has been repeated 3 times. Straight lines represent a guide to the eye.

Cell viability and growth has been recorded as a function of post deposition time up to 8 hours. The measurements were performed on 24 well plates as they contain a growth area of about 2 cm^2^ and a volume for the culture medium of 1 ml, both comparable to the electrode area and PMMA well volume of the actual transducer. The seeding densities were 1 × 10^5^ cells/ml and 1 × 10^6^ cells/ml. Each experiment was repeated three times. Figure 5a shows the cell number and Fig. 5b the measured viability as a function of postdeposition time for both seeding densities. The fully drawn curves are a guide to the eye. Cell numbers rapidly increase with time, reaching about 1 × 10^6^ cells and 4 × 10^6^ cells after 8 hours. The viability measurements show that the cells are stable over the recorded time. The average vitality of the cultures remained at all points at about 85%. We note that cell viability has been addressed on the actual transponder as well, and that comparable numbers were obtained. Therefore, glioma cells are highly viable and proliferative when their electric activity is measured.

**Figure 6:**
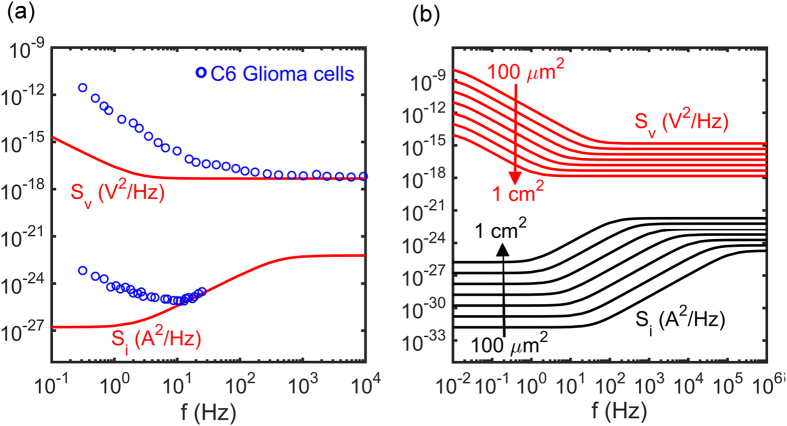
(**a**) The power spectral density of current noise, *S*_*I*_, and voltage noise, *S*_*V*_, as a function of frequency of a population of C6 glioma cells. The power spectral densities are averages over 20 consecutive unbiased measurements, of 60 s each. The electrode area was 10.5 mm^2^. The solid lines are the calculated benchmark noise spectra of the bare Au electrodes in 100 mM aqueous KCl solution, reproduced from Fig. 5. (b) Calculated benchmark current and voltage noise for Au electrode/electrolyte interfaces as a function of electrode area varied by a factor of 10 from 100 μm^2^ to 1 cm^2^. The noise is calculated from Eqs [8 and 9], assuming n equal to 1, perfect scaling with area and using fixed values of the surface charge transfer resistance, 10^6^ Ωcm^2^, the specific conductivity of the electrolyte, 200 Ωcm and a capacitance per unit area, 14 μC/cm^2^.

The glioma cells are non-electrogenic cells but do exhibit uncoordinated electrical activity. The measured signal is the sum of all the contributions of the adhered individual cells and, hence, the overall signal of the whole population appears as noise. The measured current and voltage noise is presented in Fig. 6a as a function of frequency. Measurements were performed with the same measuring electrodes. The voltage and current noise spectra were measured consecutively with a time interval between measurements of about 30 minutes. The noise spectra of the bare electrodes, *c.f.* Fig. 4, are included for comparison; for clarity we used the calculated noise spectra as they are identical to the measured noise spectra. We note that the instrumentation noise is much smaller than that of the bare electrodes. At frequencies below about 10 Hz the electrical activity of the glia cells can clearly be detected. At higher frequencies the signals cannot be distinguished from the background electrochemical noise of the electrode/electrolyte interface.

The current and voltage noise depends on the density of adhered glioma cells. When there are no cells adhered to the electrode, the spectral density of the noise is equal to that measured with only electrolyte solution. However, when cells are adhered, the noise increases. To quantify this dependence, we measured the voltage and current noise using two different seeding densities of glioma cells, *viz.* 1 × 10^5^ cells/ml and 1 × 10^6^ cells/ml. The noise was measured about two hours after seeding and each experiment was repeated three times, each experiment starting from a new batch of glioma cells.

**Figure 7:**
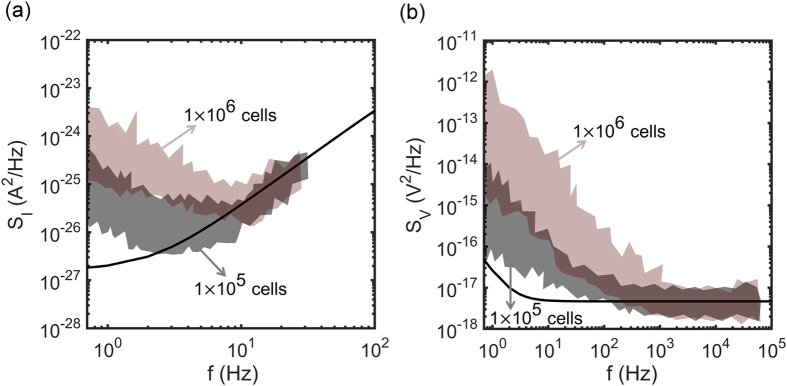
Noise spectra as a function of cell density. The current (**a**) and voltage noise (**b**) as a function of frequency. The noise was measured unbiased using two seeding densities of glioma cells of 1 × 10^5^ cells/ml and 1 × 10^6^ cells/ml. The electrode area was 10.5 mm^2^. Each spectrum is an average over 20 consecutive measurements of 60 s each. Each envelop represent three different measurements, each starting from a new glioma cell batch. The darker region in between is due to overlap between the envelopes. The solid line represents the baseline noise measured without cells, with cell culture medium only.

The power spectral density of a single experiment is an average over 20 consecutive measurements of 60 s each. The experiments were repeated three times, each experiment starting from a new batch of glioma cells. The current and voltage noise spectra of the three measurements for each seeding density are presented by the envelopes in Fig. 7a,b. The darker region is due to overlap of the envelopes. The fully drawn curves represent the baseline electrochemical noise, measured without cells, only with cell culture medium. The current noise above about 10 Hz is similar to that obtained from culture medium only measurements. At lower frequencies the current noise is larger than the baseline. Similarly, the voltage noise below about 100 Hz is larger than the baseline. Although glioma cells are reported to be non-electrogenic cells, at low frequency their activity can electrically be detected. Figure 5 shows that the density of cells adhered to the electrode increases with seeding density. Therefore the current and voltage noise increases with seeding density and is due to the presence of highly viable and proliferative glioma cell populations.

Interfaces between cells and field-effect transistors have been described as a planar electrical core-coat conductor. The cell membrane and the gate oxide are the insulating coats, and the electrolyte film in the intermediate cleft is the conducting core^25^,^26^. The so-called adhesion noise is interpreted as Nyquist noise, thermal noise through the conducting core. By taking into account the distributed nature of the cell-solid junction the frequency dependence of the power spectra density between 10 Hz and 1 MHz could be explained^27^.

Here we investigate bio-electronic signals of glioma cells adhered on Au electrodes. The noise spectra of both voltage and current below 10 Hz show a strong frequency dispersion and, therefore, cannot easily be ascribed to thermal adhesion noise. By using specific pharmacological inhibitors, it was shown that the current noise originates from opening of voltage-gated Na^+^ and K^+^ ion channels^28^. The noise shows a pronounced frequency dependence. Various power laws have been reported^29^,^30^,^31^,^32^,^33^. The contribution of voltage gated channels to the dispersion of local field potentials has not yet been identified.

The signal to noise ratio decreases with increasing frequency. At frequencies above 10 Hz, the signal is dominated by the background noise of the electrode/electrolyte interface. To illustrate the importance of the electrode impedance for the background noise we calculated the electrochemical noise as a function of electrode area. We fixed the CPE exponent *n* equal to 1, fixed the surface charge transfer resistance, the specific conductivity of the electrolyte, and the capacitance per unit area, and we assumed perfect scaling with electrode area. The calculated noise spectra are presented in Fig. 6b for circular electrodes with areas between 100 μm^2^ and 1 cm^2^. At low frequency the noise scales with the charge transfer resistance. Hence, the current noise increases linearly with area while the voltage noise decreases linearly with area. At high frequency, the charge transfer resistance is effectively shunted by the double layer capacitance and the impedance is dominated by the spreading resistance. Figure 6b shows that the background noise increases with decreasing charge transfer resistance. Hence to detect small signals, low impedance electrode interfaces are indicated. It is not straightforward to optimize the impedance of planar electrodes. Here we artificially use large area electrodes. Due to the large electrode area, single cells adhered to the electrode cannot be addressed. The spatial information is lost. The measured signal is the sum of all individual cell contributions and appears as low frequency noise. Hence this method cannot be used for measurements where a high spatial resolution is required, such as monitoring the entire range of voltages generated by a single cell. From optical micrographs we estimate that for the measurements in Fig. 5, using an electrode area of 10.5 mm^2^, we simultaneously measure the activity of about 25000 C6 glioma cells, which demonstrates that large electrodes meliorate the detection of functional biological patterns in the pico-ampere and micro-volt range such as local field potentials.

## Summary and Conclusion

We have quantitatively analyzed Au electrode/electrolyte interfaces with impedance spectroscopy. The interface has been rigorously characterized over a wide frequency range between 0.1 Hz and 1 MHz. The equivalent circuit is the charge transfer resistance in parallel with the double layer capacitance in series with the spreading resistance. The double layer capacitance is described by a constant phase element that accounts for the dispersion at low frequency. The value of the charge transfer resistance indicates that Au is an almost ideal polarizable electrode. The spreading resistance leads to a Maxwell-Wagner relaxation frequency, the value of which is determined as a function of electrode area and molarity of the aqueous KCl electrolyte solution. The electrochemical noise is measured as a function of electrode area and frequency. As the system is in thermodynamic equilibrium at zero bias, the power spectral density of the current noise and of the voltage noise unambiguously follow from the measured impedance. At frequencies below the relaxation frequency the current noise increases linearly with electrode area and the voltage noise decreases linearly with electrode area. The noise floor can be as low as 0.3 μVpp by using electrode areas up to a few mm^2^. The resulting high sensitivity is demonstrated by the extracellular detection of glioma cell populations. The minute electrical activity could be detected below a frequency of about 10 Hz in highly viable and proliferating glioma cell populations.

## Additional Information

**How to cite this article**: Rocha, P. R. F. *et al*. Electrochemical noise and impedance of Au electrode/electrolyte interfaces enabling extracellular detection of glioma cell populations. *Sci. Rep.* **6**, 34843; doi: 10.1038/srep34843 (2016).

## Figures and Tables

**Table 1:** Extracted impedance parameters by fitting the experimentally measured impedance of Fig. 1 to Eqs [3,4].

Area *S* (mm^2^)	*Q* (Ω^−1^ s^n^)	n	*R*_*ct*_ (Ω)	*R*_ct*_S (Ωcm^2^)	*C*_*dl*_ (μF/cm^2^)	*R*_*sol*_ (Ω)	*ρ* (Ωcm)
2	1.35 × 10^−7^	0.93	1 × 10^8^	1.35 × 10^6^	16.6	5.52 × 10^2^	1.76 × 10^2^
6	3.45 × 10^−7^	0.95	7 × 10^6^	2.15 × 10^5^	12.0	3.30 × 10^2^	1.82 × 10^2^
10.5	5.9 × 10^−7^	0.96	3.4 × 10^6^	1.8 × 10^5^	11.6	2.98 × 10^2^	2.18 × 10^2^

The double layer capacitance, *C*_*dl*_, is calculated using Eq. [5]. The numbers for the surface resistance, *R*_ct_*S where *S* is the area of the electrode, and capacitance per unit area have been corrected for a factor of 2 to account for the two identical electrodes used in the measurements.
